# Estimating the Temporal Domain when the Discount of the Net Evaporation Term Affects the Resulting Net Precipitation Pattern in the Moisture Budget Using a 3-D Lagrangian Approach

**DOI:** 10.1371/journal.pone.0099046

**Published:** 2014-06-03

**Authors:** Rodrigo Castillo, Raquel Nieto, Anita Drumond, Luis Gimeno

**Affiliations:** EPhysLab, Departamento de Física Aplicada, Facultade de Ciencias, Universidade de Vigo, Ourense, Spain; University of Vigo, Spain

## Abstract

The Lagrangian FLEXPART model has been used during the last decade to detect moisture sources that affect the climate in different regions of the world. While most of these studies provided a climatological perspective on the atmospheric branch of the hydrological cycle in terms of precipitation, none assessed the minimum temporal domain for which the climatological approach is valid. The methodology identifies the contribution of humidity to the moisture budget in a region by computing the changes in specific humidity along backward (or forward) trajectories of air masses over a period of ten days beforehand (afterwards), thereby allowing the calculation of monthly, seasonal and annual averages. The current study calculates as an example the climatological seasonal mean and variance of the net precipitation for regions in which precipitation exceeds evaporation (E-P<0) for the North Atlantic moisture source region using different time periods, for winter and summer from 1980 to 2000. The results show that net evaporation (E-P>0) can be discounted after when the integration of E-P is done without affecting the general net precipitation patterns when it is discounted in a monthly or longer time scale.

## Introduction

The atmospheric transport of water vapour from regions of net evaporation to regions of net precipitation is an important part of the hydrological cycle [1]. A Lagrangian approach based on the dispersion model FLEXPART [2] has been used extensively for several years to estimate sources of moisture and precipitation at both global [3], [4], [5] and regional scales [6]. These studies integrate the difference between evaporation and precipitation (E-P) to obtain the surface fresh-water flux at a monthly, seasonal or annual scale. However, when an estimation of the net precipitation wants to become separately, the evaporative term (E-P>0) in the balance of E-P is eliminated, remaining only E-P<0. The present study observes the impact of discounting the net evaporation E-P>0 term at different temporal scales from the climatological estimate of the surface fresh-water flux, using a 3-D Lagrangian approach. Suitability tests were performed using the North Atlantic region (NATL) for winter and summer seasons, for the years 1980 to 2000. The NATL source region was chosen based on the results of Gimeno et al. [3], who found this area as the dominant oceanic source providing moisture for precipitation over continents. It influences vast geographical areas, such as Eastern North America, Central America and Northern South America during JJA, and it extends its contribution also towards Europe, Northern Africa and Central South America during DJF. The influence of other large oceanic sources (e.g., Southern Indian and the North Pacific oceans) is confined towards much smaller continental areas when compared to the contribution from the NATL source. The importance of this source has been well documented in previous analysis for Central America [7], South America [8] and Europe [9]. Also the NATL source is an important oceanic contributor to the North and South American Monsoon Systems, as well as the Atlantic Inter Tropical Convergence Zone (ITCZ). It is affected by the El Niño-Southern Oscillation (ENSO) [5] and by the North Annular (NAM) modes [10]. Using the same methodology as Gimeno et al. [3], we determine the time scales for which E-P>0 may be discounted after the integration of E-P without affecting the resultant patterns of precipitation.

## Method

The present study is based on the method developed by Stohl & James [11], [12], which uses the FLEXPART Lagrangian particle dispersion model [2] and ERA-40 Reanalysis data [13] to track atmospheric moisture along trajectories through the entire depth of the atmosphere. Lagrangian particle models compute trajectories of a large number of infinitesimally small air parcels (so-called “particles”) to model the transport and diffusion of atmospheric tracers [14]. At the start of each model run, the atmosphere was “filled” homogeneously with particles, each representing a fraction of the total atmospheric mass [11]. During the run, these particles were advected using the three-dimensional Reanalysis wind, with superimposed stochastic turbulent and convective motions. The particle positions and specific humidity (q) were recorded every 6 hours. Increases (evaporation, e) and decreases (precipitation, p) in the parcel's moisture along the trajectory were calculated from changes in specific humidity (q) with time (Equation 1)
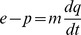
(1)where m is the mass of each particle.

Summing the moisture changes (e-p) of all of the particles in the atmospheric column over a specified area gives the surface freshwater flux (E-P), where E is the evaporation rate per unit area, P is the precipitation rate per unit area (Equation 2)
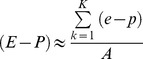
(2)where K is the total number of particles in the atmospheric column. In the present work, the global atmosphere was divided into 1.9 million particles.

Each particle is tracked for a transport time of 10 days because that is the average residence time of water vapour in the atmosphere [15]. The tracks were computed using ERA-40 Re-analysis data available at an interval of six hours (00, 06, 12 and 18 UTC), at a spatial resolution of 1° latitude by 1° longitude. All 60 vertical levels of Reanalysis data were used, from 0.1 to 1000 hPa, with approximately 14 model levels below 1500 m, and 23 between 1500 m and 5000 m.

**Figure 1:**
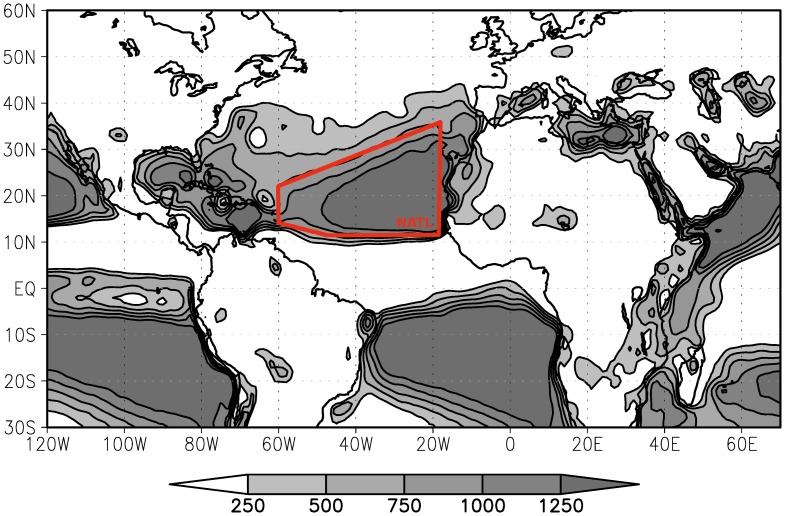
Annual vertically integrated divergence moisture flux (mm/year). Values higher than 250/year are in grey, and the interval between isolines is 250 mm/year. The North Atlantic moisture source is outlined in red. Data: ERA-40 (1958–2001).

The area of the NATL moisture source used for the forward integration of E-P was defined using a threshold of 750 mm/year for the annual divergence of vertically integrated moisture flux [3]. The spatial extent of the NATL moisture source is shown in Figure 1.

For each time step, around 30000 particles were selected over the NATL source. These particles were tracked forward in time, and the E-P field was calculated every 6 hours for ten days. Daily E-P values were calculated as the sum of the four daily outputs (at times 00, 06, 12 and 18 h), and designated (E-P)_n-day_ for the nth day (n = 1…10) of the forward trajectory. For instance, the spatial pattern (E-P)_2-day_ shows where moisture was acquired or lost during the second day of the trajectory. The total E-P integrated over the whole forward tracking period (10 days) is designated (E-P)_integrated_.

## Experiment

### Discounting net evaporation (E-P>0) at different time scales from the estimation of precipitation net (E-P<0) through surface fresh-water flux

The focus of this study is climatological continental precipitation derived solely from moisture uptake from the NATL source area. Since the Lagrangian approach is unable to separate precipitation (p) and evaporation (e) during the computation, the contribution of net evaporation was discounted by considering only negative E-P values (E-P<0). The integrated E-P values are available at different time scales, and our aim was to find time scales at which E-P>0 may be discounted without affecting the consequent climatological net precipitation patterns.

Ten-day E-P forward trajectories originating in the NATL source region were calculated for winter (December to February, DJF) and summer (June to August, JJA) for each year from 1980 to 2000. E-P>0 values were discounted at five different time scales before calculating climatological seasonal means of E-P<0. The quality of the process was assessed by evaluating differences in the mean, variance fields, correlations and using a Student-t test.

**Figure 2:**
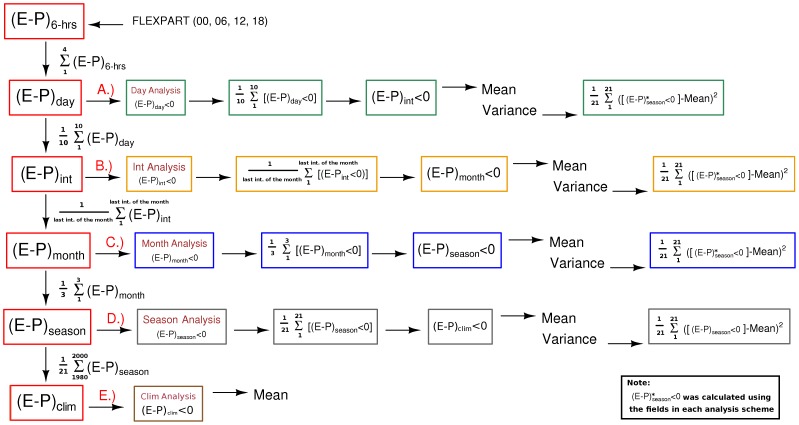
Approaches used to estimate the seasonal climatological mean and the seasonal-interannual variance of E-P<0 net through the E-P budget, by discounting the net evaporation E-P>0 from E-P at different time scales. Where the analysis abbreviations mean; hrs/hours, day/daily, int/integrated, month/monthly, season/seasonal and clim/climatological.

Figure 2 summarises the different approaches used, which were:

- ‘Daily analysis’ – (E-P) >0 values were discounted on each individual forward day of the 10-day trajectories. So, the field contained only negative values (E-P<0, net precipitation) at the beginning of the calculation. The seasonal average and the interannual variance are then computed for the period of analysis;
- ‘Integrated analysis’ – (E-P) >0 values were discounted after calculated the 10-day forward trajectories. What means that the E-P is averaged over the ten forward days, obtaining a day-integrated approximation, then the positive (E-P) values (net evaporation) are removed from the field in order to calculate the seasonal mean and the interannual variance;
- ‘Monthly analysis’ – (E-P) >0 values were discounted after calculated the monthly (E-P) mean fields. What means that firstly we calculate the monthly means of the 10-day integrated (E-P) values, and after we remove positive (E-P) values (net evaporation) from the monthly fields. Then, the monthly means of the negative (E-P) values are used to calculate the seasonal mean and the interannual variance of the negative (E-P) fields;
- ‘Seasonal analysis’ – (E-P) >0 values were discounted after calculated the seasonal (E-P) mean fields. Firstly, we calculate ‘n’ forward days, day-integrated, monthly means, and then seasonal-annual means. Then, we remove the positive (E-P) values (net evaporation) from the seasonal-annual mean fields in order to calculate the seasonal mean and the interannual variance of the negative (E-P) fields;
- ‘Climatological analysis’ – We calculated the 21-year climatological seasonal (E-P) mean, and then the positive (E-P) values were removed from the field at the end of the procedure.

For each scheme (except for “Climatological analysis”), after E-P>0 was discounted, the seasonal-annual averages of E-P<0 were computed for the 21 years of the study, and they were used to calculate the seasonal-interannual variance.

## Results

### Comparison of patterns generated by discounting E-P>0 at different time scales

**Figure 3:**
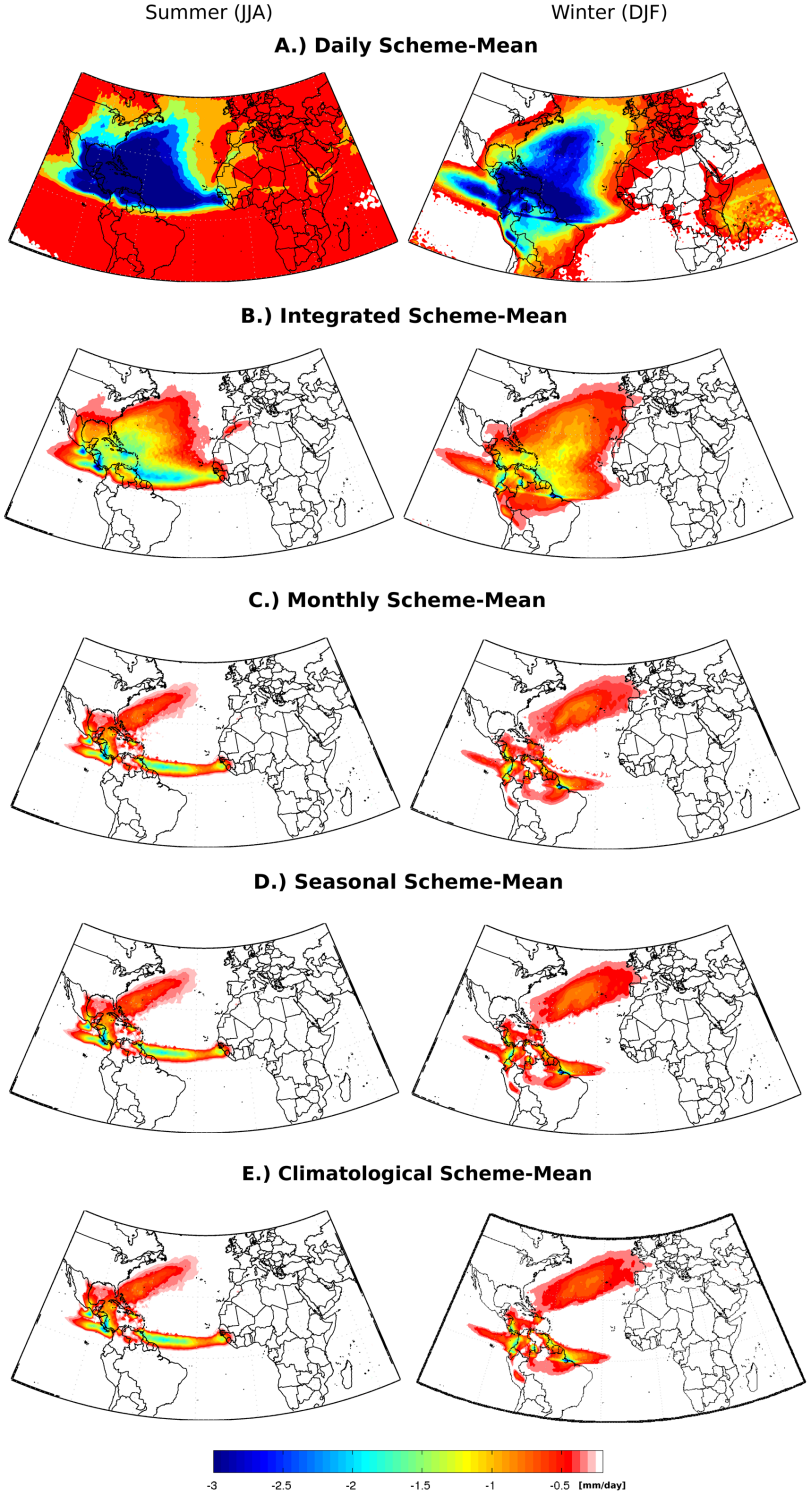
Summer (JJA) and winter (DJF) climatological seasonal net precipitation (E-P<0), from 1980 to 2000, estimated by integrating E-P over 10-day forward trajectories from the North Atlantic moisture source region, using the numerical approaches shown in **Figure 2**.

**Figure 4:**
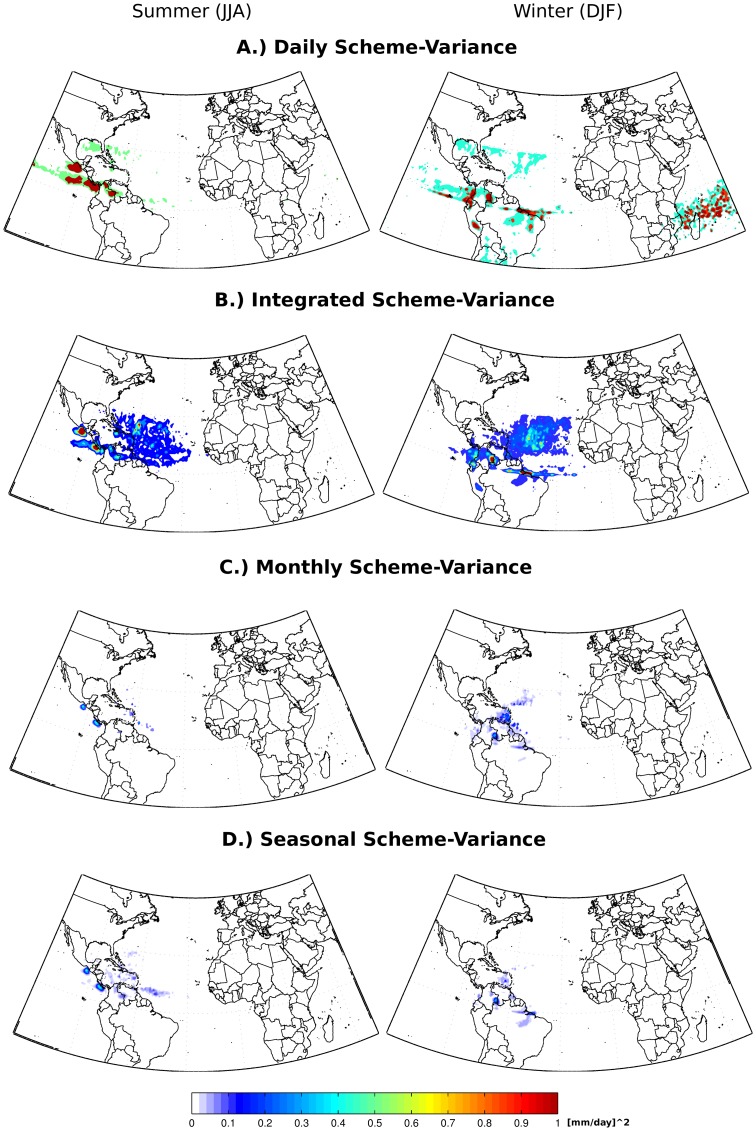
As for **Figure 3**, but for interannual variance of net precipitation (E-P<0).

The results of the suitability test, for estimating the DJF and JJA seasonal mean and interannual variance of E-P<0 for the NATL source region, are shown in Figures 3 and 4, respectively. The ‘Daily’ and ‘Integrated’ analyses overestimated mean net precipitation (Figure 3a and 3b) by comparison with the 21-year average presented by Gimeno et al. [1], and also showed high variance values (Figure 4a and 4b). By comparison, when E-P>0 was discounted at longer time scales (the ‘monthly’, ‘annual’ and ‘climatological’ schemes), the resulting seasonal climatological net precipitation patterns are similar (Figure 3c, 3d and 3e), with smaller values of interannual variance (Figure 4c, 4d and 4e).

**Figure 5:**
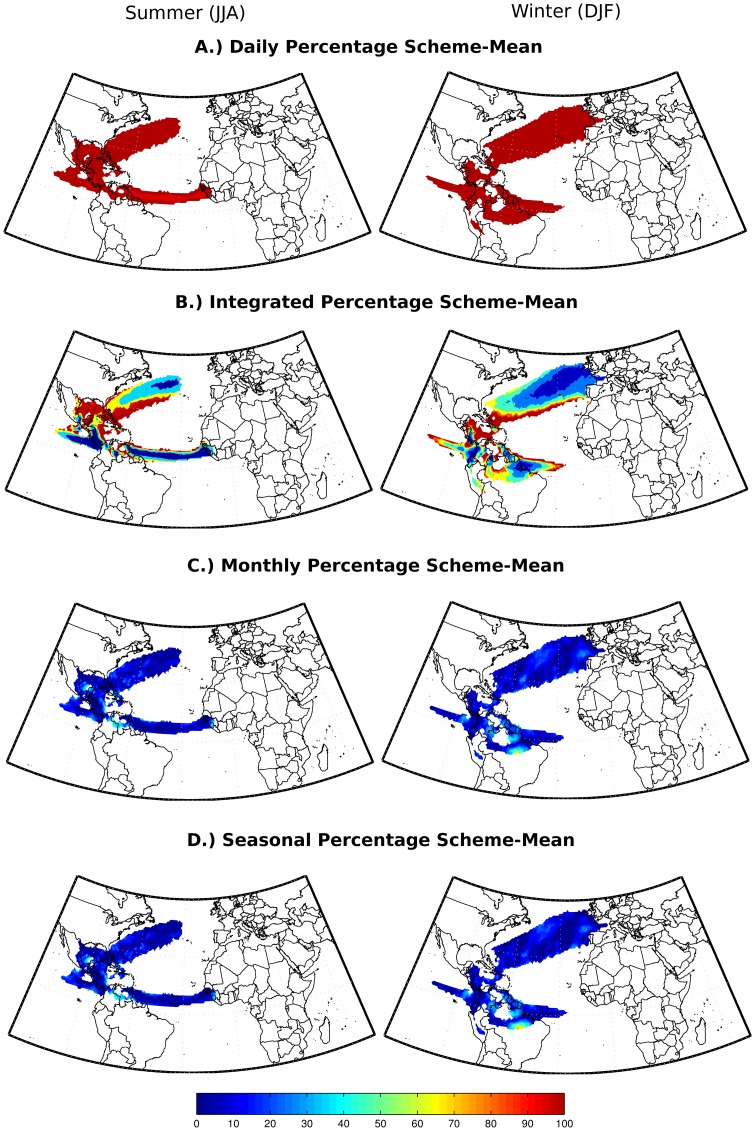
Percentage magnitude of the differences in mean net precipitation with respect to the 21-year average (climatological scheme-mean) for each one of the approaches.

In order to quantify the magnitudes of the differences in DJF and JJA mean net precipitation, we calculated the percentage error between each one of the approaches with respect to the 21-year average (climatological scheme-mean). The values of the percentage errors reduce at ‘Monthly’ or longer time scales (Figure 5).

**Figure 6:**
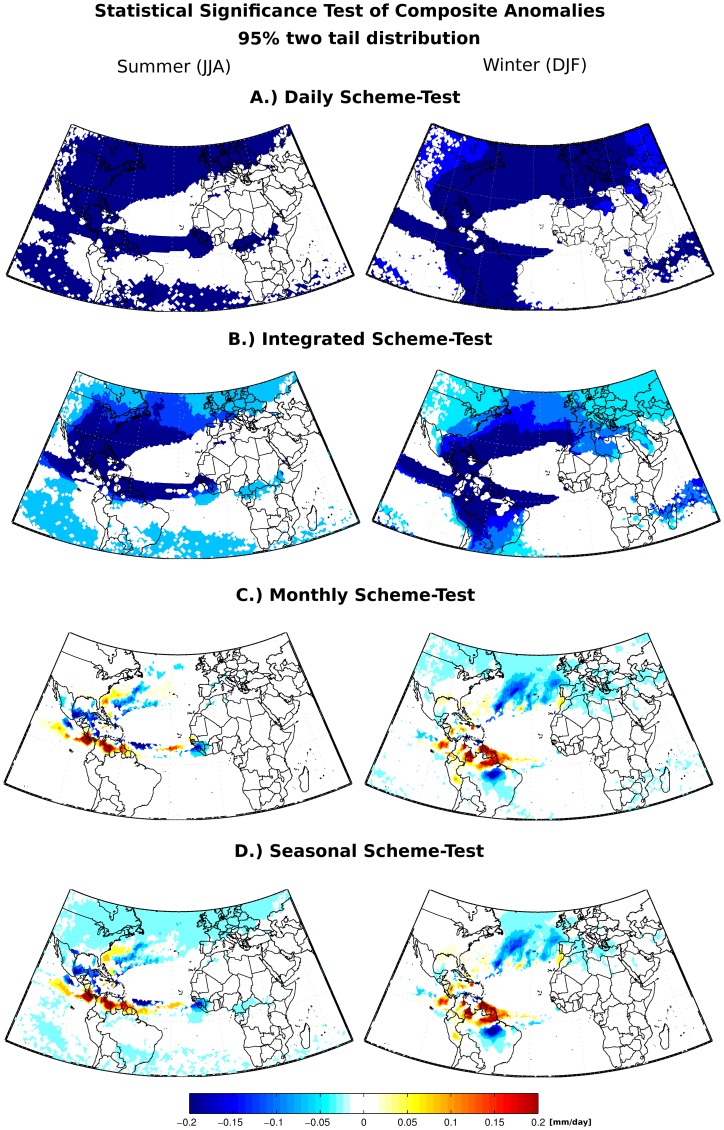
Differences between the climatological scheme-mean net precipitation and the shorter time scale scheme-means. Only significative differences through the T test using a two tail distribution and 95% of significance are shown.

**Figure 7:**
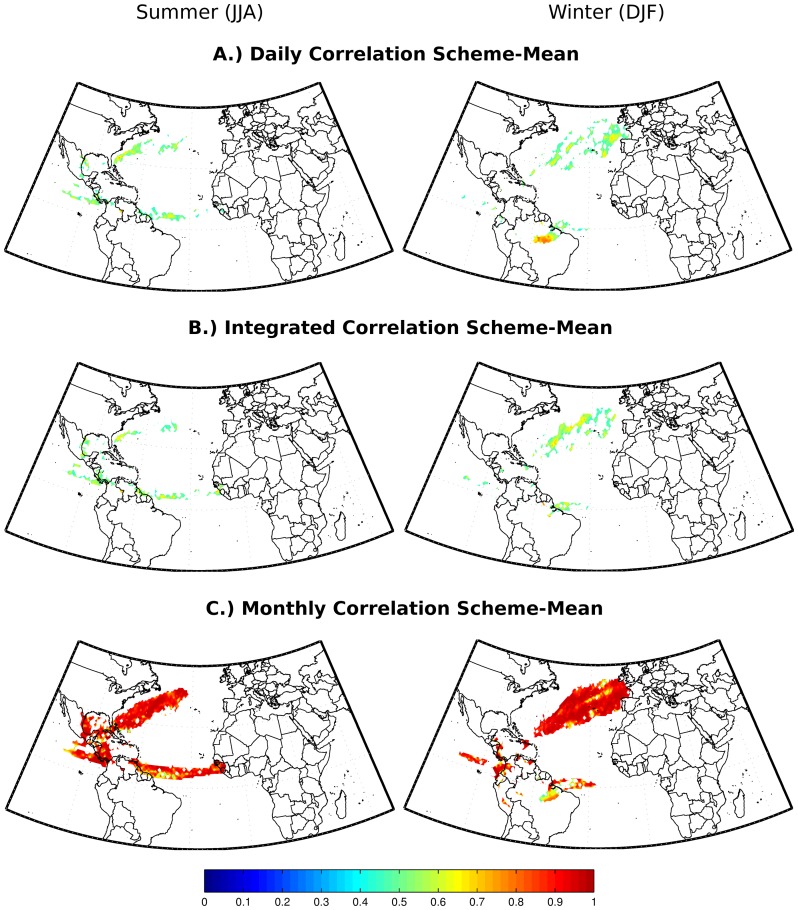
Pearson correlation of the 21-year of the net precipitation between the seasonal scheme-mean and the shorter time scale scheme-means (T test with 95% of significance).

Two statistical tests were performed. The first one evaluates the significance of the differences between the climatological scheme-mean and the other shorter time scale scheme-means through the one sample T test using the two tail distribution with 95% of significance (Figure 6). This test revealed that the differences between the means reduce at ‘Monthly’ and the longer time scales. The second one is the calculation of the Pearson time correlation coefficients (and the respective T statistical test with the 95% level of significance) of the 21-year net precipitation values obtained through the seasonal scheme-mean and the other ones estimated via the shorter time scale schemes. The main finding is that at ‘Monthly’ time scale the resulting correlation displays a very similar pattern with respect to the seasonal one, suggesting that both schemes reproduce quite similar patterns of the interannual variability (Figure 7).

Figures 5, 6 and 7 corroborate our conclusions that it is possible to discount (E-P) > 0 values after the integration of (E-P) without affecting the general net precipitation patterns, if the positive (E-P) values are discounted in monthly or longer time scales. It is likely that discounting (E-P) >0 values at shorter time scales (‘Daily’ and ‘Integrated’ analyses) distorts the long-term atmospheric column moisture budget, leading to overestimated moisture losses and expanded sink regions, as shown in the climatological averages in Figures 3a and 3b.

## Conclusions

The aim of the present study was to evaluate the impact of discounting net evaporation at different temporal scales, when estimating the climatological seasonal precipitation using the atmospheric component of the E-P moisture budget. Suitability and quality tests were performed using 3-D Lagrangian approach data by forward tracking from the North Atlantic moisture source, during winter and summer seasons from 1980 to 2000. Discounting E-P>0 from the E-P budget was tested at five different time scales, and the corresponding climatological seasonal E-P<0 means and interannual E-P<0 variances were calculated.

The results show that E-P>0 can be discounted after E-P has been integrated without altering the general patterns of net precipitation, if E-P>0 is discounted using a monthly or longer time scale.
